# A randomised controlled trial of expressive arts-based intervention for young stroke survivors

**DOI:** 10.1186/s12906-020-03161-6

**Published:** 2021-01-06

**Authors:** Caitlin Kar Pui Chan, Temmy Lee Ting Lo, Adrian Ho Yin Wan, Pamela Pui Yu Leung, Marco Yiu Chung Pang, Rainbow Tin Hung Ho

**Affiliations:** 1 Centre on Behavioral Health, The University of Hong Kong, Pokfulam, Hong Kong; 2 Department of Social Work & Social Administration, The University of Hong Kong, Pokfulam, Hong Kong; 3 The Hong Kong Society for Rehabilitation, Lam Tin, Hong Kong; 4 Department of Rehabilitation Sciences, The Hong Kong Polytechnic University, Hung Hom, Hong Kong

**Keywords:** Young stroke survivors, Expressive arts-based intervention, Holistic approach, Stroke rehabilitation, Salivary cortisol

## Abstract

**Background:**

Stroke causes lasting brain damage that has numerous impacts on the survivor’s physical, psychosocial, and spiritual well-being. Young survivors (< 65 years old) tend to suffer more because of their longer overall survival time. Expressive arts-based intervention is considered a holistic approach for stroke rehabilitation because it allows participants to express their thoughts and emotions through the arts. The group environment also promotes mutual support among participants. The creative art-making process helps expand participants’ creativity and imagination as well as promote a sense of aesthetic appreciation. Previous studies have shown the effectiveness of the arts-based intervention in managing stroke and its psychosocial-spiritual comorbidities. Nevertheless, a systematic study has not been conducted, including in young survivors. This trial plans to investigate the effectiveness of an expressive arts-based intervention on bio-psychosocial-spiritual outcomes in young Chinese stroke survivors.

**Methods/design:**

A single-blind, two-arm cluster randomised control trial with a waitlist control design will be adopted. One hundred and fifty-four stroke survivors, aged 18–64 years with modified Rankin Scale scores of 1–4, will be screened and randomised to either an expressive arts-based intervention group or a treatment-as-usual waitlist control group. The intervention group will receive a 90-min session once a week for a total of 8 weeks. All participants will be assessed three times: at baseline, 8 weeks, and 8 months after the baseline. Study outcomes include measures of depression and anxiety, perceived stress, perceived social support, hope, spiritual well-being, quality of life, salivary cortisol, blood pressure, and heart rate.

**Discussion:**

This study is expected to contribute to the current knowledge on the effectiveness of an arts-based intervention on the holistic wellness of young stroke survivors. The findings will help stroke survivors and healthcare professionals make better choices in selecting practices that will yield maximum benefits, satisfaction, adherence, and sustainability. In addition, the examination of the relationships between bio-psychosocial-spiritual variables will help contribute to the development of holistic care for the survivors.

**Trial registration:**

ClinicalTrials.gov, NCT03729648. Registered 31 October 2018 - Retrospectively registered, (329 words)

**Supplementary Information:**

The online version contains supplementary material available at 10.1186/s12906-020-03161-6.

## Background

Stroke causes lasting brain damage that impairs several body functions, resulting in restrictions in all aspects of life [1]. It does not merely affect one’s independence but also one’s psychosocial well-being and quality of life [2–11]. Young stroke survivors (< 65 years old) are more likely to suffer to a greater extent than their older counterparts because of their longer overall survival time. They often report unmet psychosocial-spiritual needs [7, 10, 12–18]. To help young stroke survivors address their unique psychosocial-spiritual concerns, which have been found to be crucial for rehabilitation adherence and achieving favourable recovery outcomes [11, 19], there is a need for a holistic rehabilitation programme, particularly the non-pharmacological one, as an adjuvant to conventional physical and occupational rehabilitation to buffer against mental health issues, reduce psychosocial stress, resume social connections, and re-instill hope [6, 8, 20–25].

### The need for a holistic, arts-based approach for the rehabilitation of young stroke survivors

Arts-based intervention refers to the application of the arts making process in a healthcare setting to deliver a novel and creative experience for therapeutic purposes [26]. The therapeutic use of arts has been considered as a way to promote holistic wellness [27]. During the process, the use of different art modalities (e.g. music, dance, visual art, drama, writing, etc.) may stimulate different parts of the brain (e.g. visual, auditory, tactile, etc.) [28]. Also, the multi-sensory stimulations created by the colours, shapes, textures, patterns of tone and rhythm, and quality of body movements and gestures, etc. would play an important role in facilitating emotional expression, abstract thinking, and/or personal reflection [29]. Through exploring the relationship between these bodily sensations and one’s thoughts and feelings, the person may uncover strengths, gain insight, and reclaim aspects of self-identity and worthiness of life [30]. Moreover, creating arts contributes to feelings of autonomy and dignity when other aspects of life seem out of control [31]. It also allows survivors to move away from illness-related preoccupations and derive greater satisfaction and self-esteem as they witness the quality of their artwork and gain positive feedback from others [32]. The non-verbal communication and symbolic expression through art media during the art-making process can further enhance engagement and help express feelings or thoughts that are difficult to verbalize. An expressive arts-based intervention is uniquely equipped to take advantage of utilising all art modalities, such as visual art, music, dance/movement, drama, and writing [33], to address the psychosocial-spiritual needs of stroke survivors [18]. Nevertheless, systematic research on its effectiveness is still limited, including on young survivors [27, 34, 35].

### Salivary cortisol levels and stroke

Salivary cortisol is a stress biomarker and an objective measure of psychological stress that reflects the functioning of the hypothalamic-pituitary-adrenal axis. A previous study showed that cortisol levels were elevated in patients who had undergone more severe strokes, and such distortion in the diurnal cortisol profile is linked with a longer hospital stay for inpatients, higher dependency, and a greater risk of depression and delirium in outpatient stroke survivors [36]. Given the important implications of psychological distress and elevated cortisol levels on stroke severity and rehabilitation outcome, it is important to examine the changes in psychological distress and cortisol profiles during the rehabilitation period.

### Research objectives

The proposed study will primarily examine the effectiveness of an expressive arts-based intervention on bio-psychosocial-spiritual outcomes in young Chinese stroke survivors in comparison with the outcomes in the treatment-as-usual control group across different time points. It will also explore the associations between the psychosocial-spiritual variables and cortisol profiles as well as the changes in those associations across time. Moreover, participants’ nature of ischaemic or haemorrhagic stroke will be accounted for using statistical methods to explore the common and differential effects of the expressive arts-based intervention on both types of stroke.

## Methods/design

### Research design

**Fig. 1:**
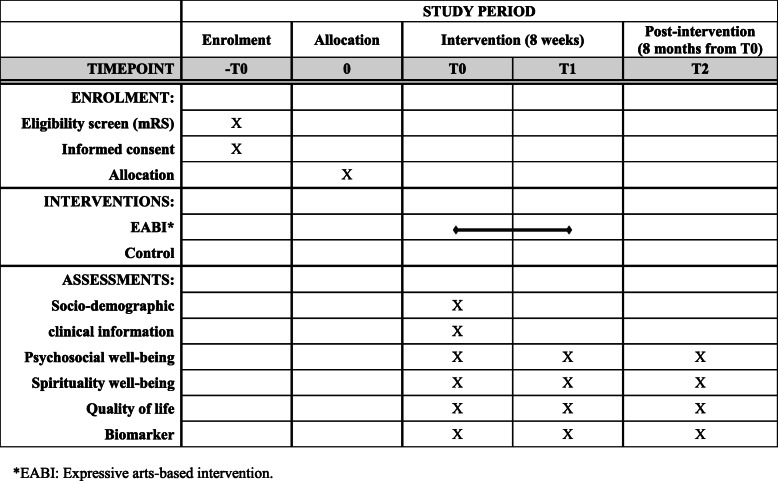
Schedule of enrolment, interventions, and assessments. *EABI: Expressive arts-based intervention

**Fig. 2:**
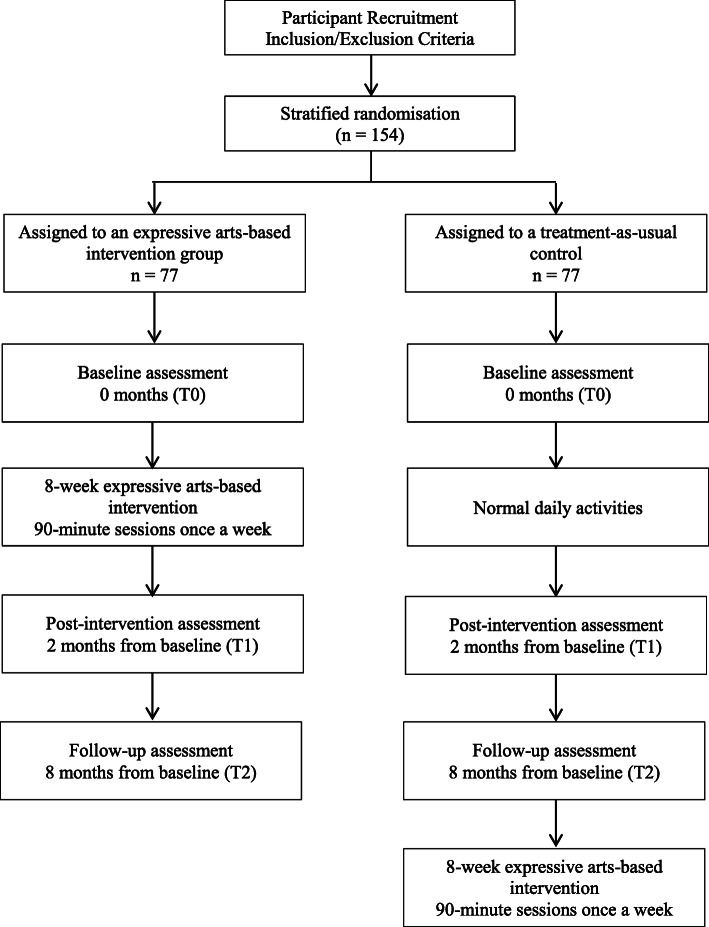
CONSORT diagram of the intervention and waitlist control groups and data collection points

The study will adopt a single-blind, two-arm cluster randomised control trial design with treatment-as-usual control. It will comprise an eight-week intervention phase and a six-month maintenance phase. The bio-psychosocial-spiritual well-being of participants will be assessed three times: at baseline (T0), 2 months after the baseline (post-intervention, T1), and 8 months after the baseline (T2) (see Fig. 1). The study design, adhering to CONSORT guidelines, is summarised in Fig. 2.

### Participant eligibility and recruitment

Participants will be recruited via referrals from public hospitals, Community Rehabilitation Network (CRN), Community Rehabilitation Day Centres (CRDC), and Patient Self-help Groups in Hong Kong, Department of Rehabilitation Sciences of The Hong Kong Polytechnic University, and Faculty of Education (Division of Speech and Hearing Sciences) of the University of Hong Kong. Promotional materials will also be delivered through social media and newspapers. A modified Rankin Scale (mRS) assessment will be performed by a trained research coordinator to screen for non-bedridden survivors who are living with post-stroke symptoms (mRS scores of 1–4). Informed consent will be obtained from the eligible participants who fulfil the inclusion and exclusion criteria.

#### Inclusion criteria

- Presence of a single-lesion stroke in the left or right, temporal, frontal, parietal, or subcortical brain region
- Experience of a major stroke event within the last 60 months from the time of study participation
- Diagnosis of either (a) ischaemic or (b) haemorrhagic stroke
- Disability grade 1 to 4 on mRS
- Residual function of the affected extremity
- Ability to understand instructions, both verbal and written, in Chinese
- Aged 18–64 years

#### Exclusion criteria

- Concurrent diagnosis of major medical or psychiatric disorders other than stroke
- Currently receiving hospital treatment and care
- Presence of hearing or visual deficits, even with aids
- Total paralysis of the upper limbs
- Amputation of one of the limbs

### Randomisation and stratification

The consenting participants will be randomised into an expressive arts-based intervention group or a treatment-as-usual control group on a 1:1 basis. The randomisation will be stratified by sex and degree of disability to balance the covariate effect across the two arms. Each participant will be assigned a computer-generated, random non-repeating number (from 1 to 200). They will then be ascendingly sorted into each cluster with the top half of the list (small numbers) being assigned to the intervention group and the rest being assigned to the control group.

### Intervention

Besides routine healthcare and rehabilitation services, the intervention group will receive an expressive arts-based intervention that consists of eight 90-min sessions, once per week with a total of 12 contact hours. The intervention will be delivered at the Centre on Behavioral Health, The University of Hong Kong, and the CRDC, in a small group format of 6–8 participants. A registered expressive arts therapist will be recruited to conduct all the intervention groups after receiving training from the Principle Investigator (PI) of the project (corresponding author). Treatment fidelity and quality will also be monitored by the PI in the form of regular on-site and off-site supervision throughout the project. The structure and content of the intervention (see Table 1) are informed by the literature on arts-based interventions and the holistic needs of various Chinese clinical populations [37–39]. Different concepts regarding psychosocial-spiritual well-being (e.g. body-mind connection, resilience, stress, and hardship in life, internal and external resources, and hope) will be explored and discussed in different sessions. Each session will be started by greeting the participants and introducing the session theme. Somatic exercises will be used to warm-up the participants physically and psychologically. Subsequently, the participants will be guided to engage in an art creation process, which aims to facilitate the expression of their thoughts and feelings in relation to the specific theme of the session. After that, they will be encouraged to share their artworks and art-making experiences. By articulating the relationships between the arts, the creative process, and the situations encountered in real life, the participants may develop a deeper understanding of self as well as new insights. The session will be ended with a closing ritual to consolidate experiences and build group cohesion. To cater to the participants’ different degrees of disability, activities will be performed in standing or sitting positions as per need. The structure of each session will be kept similar, but the content will be appropriately adjusted to suit the immediate needs and dynamics of the group.

**Table 1:** Structure, themes and objectives of the intervention

Phases	Themes	Objectives
Phase 1: Empowerment and rapport building	Session 1: Greetings and Imagination	- Build up relationships and enhance imagination through arts
	Session 2: Body-mind connection	- Raise awareness on body-mind connection
	Session 3: Creativity	- Enhance capacity and flexibility for problem-solving through the use of creativity
Phase 2: Resilience strengthening	Session 4: Body senses	- Facilitate emotional awareness and expression through bodily felt senses
	Session 5: Stress in daily life	- Release and transform stress in daily life
	Session 6: Adversities in life	- Allow expression and transformation of feelings in relation to the challenges caused by stroke
Phase 3: Consolidation and hope restoration	Session 7: Treasures in life	- Identify key resources sustaining them throughout the course of recovery
	Session 8: Review and celebration	- Review and consolidate the group experience - Celebration and farewell

The treatment-as-usual control group will continue with routine healthcare and rehabilitation services. They will be given the option to participate in the expressive arts-based intervention after the eight-month study period.

### Blinding

Owing to the nature of the trial, the participants and therapist cannot be blinded to the allocation. The data analyst and the researcher who will conduct the laboratory assessment will be blinded by recoding the demographic and group information during the process of data entry.

### Study outcomes

Measures of the study outcomes pertain to areas of bio-psycho-social-spiritual well-being of stroke survivors, including (a) psychosocial and spiritual well-being, (b) stroke-related quality of life, (c) physiological biomarkers, and (d) demographics and clinical details. mRS, blood pressure, and heart rate will be assessed by the research coordinator. Other measures will be completed by the participants themselves at all study time points. To promote participant retention, cash coupons will be given to participants who complete all the evaluation exercises.

#### Screening instrument

##### Disability

mRS is a clinician-reported rating scale for measuring the degree of disability of stroke patients. The scale is rated on an ordinal scale of 0 to 6 with a higher score denoting a higher level of post-stroke disability [40].

#### Psychosocial assessments

##### Depression and anxiety

The Chinese version of the Hospital Anxiety and Depression Scale [41] will be used to capture the anxiety and depressive symptoms. The 14-item, 4-point scale measures anxiety (7 items) and depression (7 items) subscales. The measure will be used as an outcome measure on mental health distress across time.

##### Perceived stress

Perceived stress will be assessed by the Chinese Perceived Stress Scale [42]. Using a 5-point Likert scale (0–4), the scale consists of 10 items about the degree to which life events are appraised as stressful.

##### Perceived social support

Perceived social support will be captured by the Chinese version of the Multidimensional Scale of Perceived Social Support [43]. The 12-item scale is composed of subscales for perceived social support from the family, friends, and significant others rated on a 7-point Likert scale.

#### Spirituality well-being assessments

##### Hope

The Chinese version of the Adult State Hope Scale [44] will be used to measure hope. The 6-item scale, rated on an 8-point scale, yields an aggregate score of hope as well as the agency and pathway subscales.

##### Spiritual well-being

Participants’ intention to find peace and to take care of their own spiritual needs will be measured by the 3-item spiritual care subscale of the Body-Mind-Spirit Holistic Well-being Scale rated on an 11-point anchored scale. The scale measures different dimensions of subjective well-being in the context of afflictions and equanimity [45].

#### Quality of life assessments

##### Stroke-specific quality of life

The Chinese version of the Stroke-specific Quality of Life (SS-QoL-CH) [46], Short Form is a 12-item disease-specific health-related quality of life measure widely applied in stroke research. The scale yields two quality of life subscales in physical and psychosocial components rated on a 5-point scale.

##### Health-related quality of life

The Chinese 12-item Short Form (SF-12) Health Survey [47] will be used to measure the health-related quality of life on the dimensions of physical and emotional well-being.

#### Biomarker

##### Salivary Cortisol

Saliva samples will be collected at five prescribed time points (awakening, 45 min post-awakening, noon, 5 pm, and 9 pm) using the Salivette kit (Sarstedt; Nümbrecht), which includes a cotton swab to place under the tongue. A trained research coordinator with prior experience in using the kit will explain the collection procedures to the participants in detail. A record sheet will also be included in the package to document the participants’ health behaviours and activities on the day of saliva collection that might affect the diurnal cortisol rhythm, including (a) smoking habit, (b) consumption of alcohol/coffee on that day, and (c) subjective evaluation of sleep quality, total sleep duration, and stress levels on a scale of 1 to 10. All of these measures may affect the diurnal cortisol rhythm and will be controlled in the analysis.

##### Blood pressure and heart rate

Blood pressure and heart rates will be taken following the guidelines of the Canadian Medical Association [48]. They will offer basic information on the health condition of the cardiovascular system, e.g. hypertension situation of the participants, which may relate to the risk of stroke. The parameters will be measured twice from each arm with a 5-min rest interval between measurements. The collected readings will be averaged.

### Socio-demographic and clinical information

#### Demographics

Socio-demographics, such as age, gender, education level, employment and financial status, and marital status, will be documented based on self-report questionnaires.

#### Clinical data

Participants’ clinical profiles, including types of stroke, time-lapse from the first occurrence, onset and history of psychiatric disturbance, presence of comorbidity if any (such as physical disabilities, hypertension, diabetes mellitus, or any other form of vascular disease), treatment and medication record, and psychosocial support and/or rehabilitation service utilisation will be documented.

### Data safety and monitoring board

The General Research Fund of the Research Grants Council and the Institutional Review Board will monitor the safety and progress of the study. Progress reports will be submitted to these two institutes at 18- and 12-month intervals, respectively.

### Data management

Data will be entered into a database immediately after data collection from each participant at each time point by the research coordinator. Upon the completion of data entry, data screening will be conducted by the data analyst. Any invalid input will be double-checked and/or re-entered by another research team member to ensure the data is reliable and valid for later analysis.

### Sample size estimates

To achieve a statistical power of 80% with a medium effect size (Cohen’s d = 0.63) at a significance level of 0.05 in regression modelling (latent growth modelling) under the proposed two-arm, three-time point design, a sample size of 116 is needed according to Monte Carlo simulation. Assuming an attrition rate of 25% based on prior trials of arts-based therapies in stroke survivors [49], a total of 154 participants will be required (i.e. 77 per arm).

### Statistical analyses

#### Exploring the effectiveness of expressive arts-based intervention

Intention-to-treat analysis will be used to maintain the prognostic balance resulting from randomisation. Full information maximum likelihood will be conducted to estimate the missing data. Analysis of variance and Chi-square independence tests will be performed using the Statistical Package for Social Sciences (SPSS) (IBM; New York) to compare the demographic profile of the two groups. Latent growth modelling in Mplus will be used to explore the effectiveness of the intervention over the assessment points and in comparison to the control treatment.

#### Analysis of salivary cortisol

Saliva samples will be centrifuged at 3000 rpm for 15 min at room temperature. The concentration of cortisol in each sample will be calculated using the Salimetrics Salivary Cortisol ELISA kit (Salimetrics, LLC; Carlsbad). The assay sensitivity is 0.193 nmol/L, and the intra- and inter-assay coefficients of variation are 3 and 10%, respectively. The mean cortisol level across the day, the total cortisol level indexed by the area under the curve, and the diurnal cortisol slope will be calculated.

To explore individual trajectories of changes in cortisol levels over time and the complex relationships between different variables, a two-level individual growth curve model using Mplus software will be adopted as cortisol measures at five daily time points are nested within the participants. The method is an appropriate variant of multiple regression modelling for the nested structure of cortisol data.

#### Common and differential effects of the intervention on ischaemic and haemorrhagic stroke survivors

To explore the potential differential effects of the intervention on ischaemic and haemorrhagic stroke survivors, treatment effects on the two groups will be directly compared in multi-group conditional growth models.

### Study organisation and funding

The study is funded by the General Research Fund of the Research Grants Council (GRF/HKU/17609417). The trial and the expressive arts-based intervention will be coordinated and conducted by the Centre on Behavioral Health, the University of Hong Kong.

## Discussion

Stroke rehabilitation is a race against time. The clinical popularity of the use of the arts in stroke rehabilitation calls for the need for rigorous research evidence on their benefits. Nevertheless, a systematic study related to arts-based intervention has not yet been conducted, including among younger stroke survivors, who may experience greater and longer-term impacts in the psycho-physiological and social-spiritual aspects. This study is expected to contribute to the current knowledge on the effectiveness of arts-based rehabilitation on young stroke survivors. Both psychological and physiological outcomes will be examined for a comprehensive understanding of the biological, psychological, social, and spiritual changes after participating in a non-pharmacological, engaging, safe, and enjoyable multi-modal expressive arts-based intervention for rehabilitation. The findings will help stroke survivors and healthcare professionals make better choices in selecting practices that will yield maximum benefits, satisfaction, adherence, and sustainability for young stroke survivors. In addition, the examination of the relationships between bio-psychosocial-spiritual variables may help understand the complex relationships between these factors after stroke and during rehabilitation, which will contribute to the development of holistic care for the survivors.

### Ethics and dissemination

#### Research ethics approval

The safety and ethical conformity of the study have been reviewed and approved by the Human Research Ethics Committee of the University of Hong Kong (EA1702058) and the Institutional Review Board of the University of Hong Kong/Hospital Authority Hong Kong West Cluster (UW18–467) and East Cluster (HKECREC-2019-111). The study is also registered with the ClinicalTrials.gov Registry (NCT03729648).

#### Protocol amendments

Amendments to the protocol, including changes to inclusion criteria, recruitment, or data collection procedures, will be agreed upon by the principal investigator and approved by the Human Research Ethics Committee of the University of Hong Kong, the Institutional Review Board of the Hospital Authority, and ClinicalTrials.gov before implementation.

#### Consent

The study details and requirements will be fully described and explained to the eligible participants by a research coordinator before obtaining informed consent from them (see Appendix I).

#### Confidentiality

Any information obtained in this study will remain strictly confidential and be used for research purposes only. Codes, not names, are used on all reports and publications related to this study to protect confidentiality. All collected questionnaires will be kept in locked cabinets and saliva samples will be stored at a locked laboratory freezer in the Centre on Behavioral Health at the University of Hong Kong. The electronic dataset will be stored in encrypted computer storage. Data containing personal identifiers will be kept for a maximum of 3 years after the publication of the first paper upon the end of the study.

#### Declaration of interests

The authors declare that there is no conflict of interest regarding the publication of this protocol.

#### Access to data

Only the research team members of this project will have access to the data.

#### Ancillary and post-trial care

There are no provisions for ancillary or post-trial care.

#### Dissemination policy

Manuscripts resulting from this trial will be published in academic journals and/or abstracts of papers will be presented in academic conferences. Authorship eligibility includes (1) substantial contributions to the study design or the process of data acquisition, analysis, or interpretation, (2) drafting or revising the manuscript, and (3) approving the final manuscript. There is no intention to use professional writers.

### Supplementary Information

**Additional file 1.**

## Data Availability

Not applicable. This is a study protocol, no datasets are generated at the present.

## Appendix

**Department of Social Work and Social Administration, The University of Hong Kong.**

**Centre on Behavioral Health, The University of Hong Kong.**

**The Psycho-physiological & Social-Spiritual Effects of Expressive Arts-based Intervention on Young and Pre-elderly Stroke Survivors: A Randomized Controlled Study.**

**Informed Consent Form.**

Evidence has emerged to support the effectiveness of different arts modalities in improving the psycho-physiological and social-spiritual well-being of young and pre-elderly stroke survivors. You are invited to participate in a research study conducted by Prof. Rainbow T.H. Ho, of the Department of Social Work and Social Administration, The University of Hong Kong, and Centre on Behavioral Health (The University of Hong Kong). The primary purpose of the research is to investigate the short and long-term psycho-physiological and social-spiritual effects of an Expressive Arts-based Intervention on younger and pre-elderly Chinese stroke survivors.

**Research Procedures.**

All eligible participants will be randomly assigned to either the Expressive Arts-based Intervention Group or the Treatment-As-Usual Control Group upon screening for inclusion-exclusion criteria. You will be invited to complete an assessment package (including questionnaires and a two-day saliva cortisol kit) at 3 time points including the baseline, post-intervention (8-week time point), and at 6-month post-intervention. Participants in the Expressive Arts-based Intervention Group will receive an 8-week expressive arts-based program (8 sessions, 90 min per session) provided by a trained expressive arts therapist, while the participants in the control group will continue with routine healthcare services, and they will be given the option to take part in the same intervention program upon completion of the 6-month post-intervention follow-up assessment. Some of the participants will also be invited to join a 45 min in-depth interview at post-intervention (8-week time point), and at 6-month post-intervention. The interview will focus on the changes after the onset of stroke and the experiences of participating in the Expressive Arts-based Intervention Group. The interview will be audio-recorded for data analysis. All data obtained during the study will remain strictly confidential; and all personal identifying information will be erased from the database 3 years after the first publication from this study.

**Potential Risks/ Discomforts and their Minimization.**

Some of the questions in the questionnaires may involve personal information (including your medical conditions, and mental health status). The collection of saliva involves putting a small piece of cotton in the mouth which may cause slight discomfort. During the assessment process, some may feel stressful and your emotions may be temporarily affected. Nevertheless, such distress, if any, should not be greater than what we experience in everyday life. In any case, if you need help or support, please inform us or the research staff.

**Confidentiality.**

Any information obtained in this study will remain strictly confidential, will be known to no-one, and will be used for research purposes only. Codes, not names, are used on all reports to protect confidentiality. All data will be stored on an encrypted storage at the Centre on Behavioral Health; and be is only accessible by the Principal Investigator, and the research team members. Codes, not names, are used on all reports and publications related to this study. All data will be erased from the database 3 years after the first publication of this study.

Your participation in the study is voluntary; you can choose to stop at any time without negative consequences. If you have any questions about the research, you may raise them now. If you have further questions about the research, please feel to contact Prof. Rainbow T.H. Ho (Office: 2831 5158). And if you have questions about your rights as a research participant, contact the Human Research Ethics Committee, HKU (2241–5467).

I understand the procedures described above and ^**^ agree to/ do not agree to participate in this study (^**^ Please delete as appropriate).

______________________.

Signature of Participant.

______________________.

Name of Participant.

______________________.

Date.

Date of preparation:28 December 2017.

Expiration date:9 March 2021.

HREC Reference Number:EA1702058.

## Abbreviations

CRN: Community Rehabilitation Network
CRDC: Community Rehabilitation Day Centres
mRS: Modified Rankin Scale
PI: Principle Investigator
EABI: Expressive arts-based intervention
SS-QoL-CH: The Chinese version of the Stroke-specific Quality of Life
SF-12: The Chinese 12-item Short Form Health Survey
SPSS: The Statistical Package for Social Sciences
