# Supplementary material for: A randomised controlled trial of expressive arts-based intervention for young stroke survivors
Source: BMC Complement Med Ther. 2021 Jan 6;21:7. doi: 10.1186/s12906-020-03161-6 (PMC7789770; doi:10.1186/s12906-020-03161-6)
Supplement: Supplementary file 1 — Additional file 1. [file 12906_2020_3161_MOESM1_ESM.docx]

**Administrative information**

| **Data category** | **Information** |
| --- | --- |
| Primary registry and trial identifying number | ClinicalTrials.gov NCT03729648 |
| Date of registration in primary registry | 31 October, 2018 |
| Secondary identifying numbers | Nil |
| Source(s) of monetary or material support | General Research Fund of the Research Grants Council (GRF/HKU/ 17609417) |
| Primary sponsor | General Research Fund of the Research Grants Council |
| Secondary sponsor(s) | Nil |
| Contact for public queries | *Rainbow T.H. Ho*, PhD, BC-DMT, AThR, REAT, RSMT, CGP, CMA [Tel: 852-2831-5158] [Email: tinho@hku.hk] |
| Contact for scientific queries | *Rainbow T.H. Ho*  Centre on Behavioral Health, 2/F, The Hong Kong Jockey Club Building for Interdisciplinary Research, 5 Sassoon Road, Pokfulam, Hong Kong |
| Public title | A randomised controlled trial of expressive arts-based intervention for young stroke survivors |
| Scientific title | The psycho-physiological and social-spiritual effects of expressive arts-based intervention on young and pre-elderly stroke survivors: A randomized controlled study |
| Countries of recruitment | Hong Kong, China |
| Health condition(s) or problem(s) studied | Bio-psychosocial-spiritual wellness of young stroke survivors |
| Intervention(s) | Intervention arm: Expressive arts-based intervention (One 90-minute session per week for 8 weeks), plus routine healthcare and rehabilitation services Control arm: Routine healthcare and rehabilitation services |
| Key inclusion and exclusion criteria | Inclusion criteria   - Presence of a single-lesion stroke in the left or right, temporal, frontal, parietal or subcortical brain region - Experience of a major stroke event within the last 60 months from the time of study participation - Diagnosis of either (a) ischaemic or (b) haemorrhagic stroke - Disability grade 1 to 4 on mRS - Residual function of the affected extremity - Ability to understand instructions, both verbal and written, in Chinese - Aged 18–64 years   Exclusion criteria   - Concurrent diagnosis of major medical or psychiatric disorders other than stroke - Currently receiving hospital treatment and care - Presence of hearing or visual deficits, even with aids - Total paralysis of the upper limbs - Amputation of one of the limbs |
| Study type | Interventional Allocation: randomised Intervention model: parallel assignment Masking: single blind (investigator) Primary purpose: Treatment |
| Date of first enrolment | 1 March, 2018 (date of receiving the first referral) |
| Target sample size | 154 |
| Recruitment status | Recruiting |
| Primary outcome(s) | To investigate the short and long-term effects of an Expressive  arts-based Intervention for young Chinese stroke survivors on:   1. Depressive and anxiety symptoms 2. Perceived stress 3. Perceived social support 4. Hope 5. Subjective and spiritual well-being 6. Stroke-related and general quality of life, and 7. Salivary cortisol level |
| Key secondary outcomes | 1. To examine the associations between cortisol levels, diurnal cortisol profiles and psycho-social-spiritual variables in young Chinese stroke survivors. 2. To examine the changes of the associations between cortisol levels, diurnal cortisol profiles and psycho-social-spiritual variables across the time points. |

**Protocol version**

**Issue Date**: 5 November 2018
**Protocol Amendment Number**: 06

**Revision Chronology:**

| 2018-Oct-31 | Original |
| --- | --- |
| 2019-Nov-5 | Amendment No.1:   - Updated the inclusion criteria for expanding the pool of subjects eligible in participating in this research study - Updated the details about the subscale of the Chinese version of the Stroke-specific Quality of Life Short Form - Updated the masking procedures that the procedures were not double-blinded |
| 2019-Nov-22 | Amendment No. 2:   - Corrected of typographical error about the details of the subscale of the Chinese version of the Stroke-specific Quality of Life Short Form |
| 2019-Dec-11 | Amendment No. 3:   - Postponed the study start date because we experienced difficulties in recruitment. |
| 2020-May-4 | Amendment No.4:   - Updated the inclusion criteria. Subject experienced a major stroke event within 60 months from the time of study participation is eligible for the study. |
| 2020-May-28 | Amendment No.5:   - Updated the masking procedures that the procedures were single-blinded, the investigator and the outcome assessor are masked. |
| 2020-July-7 | Amendment No.6:   - Updated the masking procedures that the procedures were single-blinded, the data analyst will be blinded by having the intervention group information coded. |

**Name and contact information for the funding source**

| Funding source: | General Research Fund of the Research Grants Council |
| --- | --- |
| Address: | 7/F., Shui On Centre, 6-8 Harbour Road, Wanchai, Hong Kong SAR, People's Republic of China. |
| Telephone: | (852) 2524 3987 |
| Email: | [ugc@ugc.edu.hk](mailto:ugc@ugc.edu.hk) |

This funding source had no role in the design of this study and will not have any role during its execution, analyses, interpretation of the data, or decision to submit results.
